# Three-Dimensional Conformal Radiotherapy-Based or Intensity-Modulated Radiotherapy-Based Concurrent Chemoradiotherapy in Patients with Thoracic Esophageal Squamous Cell Carcinoma

**DOI:** 10.3390/cancers11101529

**Published:** 2019-10-10

**Authors:** Wei-Cheng Lin, Chia-Lun Chang, Han-Lin Hsu, Kevin Sheng-Po Yuan, Alexander T. H. Wu, Szu-Yuan Wu

**Affiliations:** 1 Division of Thoracic Surgery, Department of Surgery, Wan Fang Hospital, Taipei Medical University, Taipei 106, Taiwan; 101336@w.tmu.edu.tw; 2 Department of Hemato-Oncology, Wan Fang Hospital, Taipei Medical University, Taipei 106, Taiwan; 101255@mail.wanfang.gov.tw; 3 Department of Internal Medicine, School of Medicine, College of Medicine, 106 Taipei Medical University, Taipei 106, Taiwan; 4 Division of Pulmonary Medicine, Department of Internal Medicine, Wan Fang Hospital, Taipei Medical University, Taipei 106, Taiwan; 94401@w.tmu.edu.tw; 5 School of Respiratory Therapy, College of Medicine, Taipei Medical University, Taipei 106, Taiwan; 6 Department of Otorhinolaryngology, Wan Fang Hospital, Taipei Medical University, Taipei 106, Taiwan; dryuank@w.tmu.edu.tw; 7 Ph.D. Program for Translational Medicine, Taipei Medical University, Taipei 106, Taiwan; chaw1211@tmu.edu.tw; 8 Department of Food Nutrition and Health Biotechnology, College of Medical and Health Science, Asia University, Taichung 413, Taiwan; 9 Division of Radiation Oncology, Lo-Hsu Medical Foundation, Lotung Poh-Ai Hospital, Yilan 265, Taiwan; 10 Department of Radiation Oncology, Wan Fang Hospital, Taipei Medical University, Taipei 106, Taiwan; 11 Department of Radiology, School of Medicine, College of Medicine, Taipei Medical University, Taipei 106, Taiwan

**Keywords:** thoracic esophageal cancer, squamous cell carcinoma, intensity-modulated radiation therapy, concurrent chemoradiotherapy, three-dimensional conformal radiation therapy

## Abstract

Background: To date, intensity-modulated radiation therapy (IMRT) with concurrent chemoradiotherapy (CCRT) and CCRT with standard fractionation three-dimensional conformal radiation therapy (3D-CRT) have not been compared. In this study, the outcomes of IMRT-based concurrent CCRT and those of 3D-CRT-based CCRT were compared in patients with thoracic esophageal squamous cell carcinoma (TESCC). Methods: We enrolled 2062 patients with TESCC who had received CCRT and categorized them into two groups on the basis of their treatment modality: Group 1 (3D-CRT-based CCRT) and Group 2 (IMRT-based CCRT). Results: Multivariate Cox regression analysis indicated that the American Joint Committee on Cancer advanced stages (≥IIIA) and 3D-CRT were significant independent predictors of poor outcomes in patients with TESCC who received definitive CCRT. Moreover, receiving IMRT-based CCRT (adjusted hazard ratio [aHR]: 0.88, 95% confidence interval [CI]: 0.78–0.98) was a significant independent prognostic factor for overall survival (*p* = 0.0223). In Group 2, aHRs (95% CIs) for overall mortality at early (IA–IIB) and advanced clinical stages were 0.91 (0.67–1.25, *p* = 0.5746) and 0.88 (0.77–0.99, *p* = 0.0368), respectively. Conclusion: IMRT-based CCRT resulted in higher survival rates in patients with advanced clinical stages of TESCC (i.e., IIIA–IIIC), namely, clinical T3, clinical T4, or lymph node involvement.

## 1. Introduction

Esophageal cancer is the ninth leading cause of cancer death in Taiwan [1]. In Taiwan, more than 90% of patients with esophageal cancer have been reported to have squamous cell carcinomas (SCCs) [2,3,4]. The pathological types of SCC in Taiwan, China, and other Asian countries have been reported to differ from those in European, North American, and other Western countries [5,6]. While esophageal adenocarcinoma is common in Western countries, this type of cancer is not common in Asian countries [5,6]. Standard regimens of definitive concurrent chemoradiotherapy (CCRT) for esophageal cancer have been administered according to standard clinical practices in Taiwan by using the National Comprehensive Cancer Network (NCCN) guidelines, and Taiwanese oncologists have followed regimens recommended by the Intergroup (INT) 0123 (radiation therapy oncology group 94-05) phase III trial since 2002 [7,8]. The INT 0123 trial demonstrates that the treatment-related mortality rate was high in patients, with esophageal cancer, who received a high-dose of radiotherapy (RT) (64.80 Gy) [8]. The standard RT dose is 50.40 Gy for patients with esophageal cancer receiving cisplatin-based chemotherapy [8].

Esophageal squamous cell carcinoma (ESCC) is common in Asia, and patients usually die of locoregional recurrence, rather than distant metastasis [9,10,11,12,13,14,15]. In Taiwan, >90% of patients with esophageal cancer have SCC, which has a higher locoregional recurrence rate and lower survival rate than esophageal adenocarcinoma [12,13,14,15]. For patients with ESCC, improving the local control rate is necessary to reduce the mortality caused by local recurrence [9,10,11,12,13,14,15,16]. In the past 10 years, considerable advancements have led to the development of modern radiotherapy (RT) techniques, such as intensity-modulated radiation therapy (IMRT), which are associated with improved tumor coverage, high dose conformity, and reduction in the scattering of irradiation dose–volume to critical organs compared with the conventional RT technique of three-dimensional conformal radiation therapy (3D-CRT) [16,17,18,19]. However, in the initial stages of IMRT use for the treatment of malignancies, most physicians have been concerned that the use of IMRT would increase the integral dose to normal tissues and result in high radiation toxicity; consequently, IMRT use was limited [20,21]. 

Recently, an increasing number of studies have shown that IMRT for the treatment of esophageal cancer results in a lower irradiation dose–volume to the lungs and heart than conventional RT [22,23]. RT toxicities depend on the primary tumor location; thus, RT for thoracic esophageal cancer might exhibit greater scattering of irradiation doses to the lung and heart than RT for cervical or gastroesophageal (GE) esophageal cancer [24]. This is because the clinical target volume of the RT field in ESCC has been defined as the gross target volume as well as regional draining lymphatics based on the primary tumor location [25,26]. In patients with thoracic esophageal squamous cell carcinoma (TESCC), the scattered radiation dose to the lungs and heart is high; hence, the incidence of radiation-related pneumonitis, fibrosis, and cardiotoxicity is higher in TESCC than in the cervical or GE forms of ESCC [24,27,28]. In this study, we selected patients with TESCC tumors in specific locations as the analytical population in order to highlight the effects of IMRT-based CCRT on survival. 

Regimens of CCRT based on NCCN guidelines and INT 0123 are the standard treatments for ESCC in Taiwan [7,8]. However, >60% of patients included in the INT 0123 trial had early clinical stage esophageal cancer; thus, toxicity, compliance, and tolerance of CCRT might have been underestimated [8]. Moreover, in Taiwan, many patients have been diagnosed with advanced stage (IIIA-IIIC) TESCC (82.17%), and CCRT outcomes for these patients have been unsatisfactory [16]. Data from the literature have not clarified the effects of IMRT-based CCRT on survival, which has potentially reduced the irradiation toxicities to the lungs and heart and increased tumor coverage [20,23,24]. No trial has compared the outcomes of IMRT with concurrent chemotherapy (CCRT) and those of CCRT with standard fractionation 3D-CRT; consequently, the safety and efficacy of this approach, compared with standard 3D-CRT, remains undefined. This study compared the survival effects of IMRT-based CCRT and those of 3D-CRT-based CCRT in patients with TESCC who had high latent locoregional recurrence rates.

## 2. Patients and Methods

Ethics approval and consent: Our protocols were reviewed and approved by the Institutional Review Board of Taipei Medical University (TMU-JIRB No. 201402018).

Data in this cohort study were retrieved from the Taiwan Cancer Registry Database (TCRD). We enrolled patients who had received a diagnosis of TESCC between 1 January 2006, and 31 December 2014. The index date was the date of TESCC diagnosis, and the follow-up duration was from the index date to 31 December 2014. The TCRD of the Collaboration Center of Health Information Application contains detailed cancer-related information on patients, namely, clinical stage, treatment modalities, chemotherapy regimens, dose of chemotherapy, pathology, radiation modalities and doses, and treatment protocols (CCRT or non-CCRT protocols) [2,3,4]. Our study protocols were reviewed and approved by the Institutional Review Board of Taipei Medical University. The diagnoses of the enrolled patients were confirmed after reviewing their pathological data, and patients with newly diagnosed TESCC were confirmed to have no other cancer or distant metastasis.

Patients were included if they had received a diagnosis of TESCC, were ≥20 years old, and showed clinical stages IA–IIIC according to the Seventh Edition of the American Joint Committee on Cancer (AJCC) Staging Manual without metastasis. Patients were excluded if they were <20 years old or if their medical history included any of the following: Cancer before TESCC diagnosis, distant metastasis, unknown esophageal cancer sites, missing sex data, unclear staging, and non-SCC histology. TESCC was defined as ESCC with pathological evidence in the thoracic area, as indicated in the TCRD. We also excluded patients with TESCC who did not receive treatment (i.e., 3D-CRT or IMRT), received insufficient CCRT doses (<5000 cGy) after TESCC diagnosis, did not receive a cisplatin-based chemotherapy regimen, or patients who received only sequential chemotherapy and RT, chemotherapy alone, or RT alone.

We only enrolled patients who received CCRT and excluded patients who received induction chemotherapy and adjuvant chemotherapy, according to our previous studies [2,3,4]. The chemotherapy regimen was a cisplatin-based regimen (100 mg/m^2^), and each patient received at least two cycles of chemotherapy (cumulative total dose of ≥200 mg/m^2^) during CCRT [2,3,4]. Most patients with TESCC in Taiwan have received CCRT based on NCCN guidelines [7]. Therefore, patients with TESCC who received induction chemotherapy or adjuvant chemotherapy represent a minority.

IMRT is an advanced mode of high-precision RT that uses computer-controlled linear accelerators to deliver precise radiation doses to a malignant tumor or specific areas within the tumor [29,30]. We selected patients with TESCC receiving IMRT on the basis of the records in the TCRD. The application rate of IMRT in Taiwan is high [4,31,32,33,34] because it is an easily available RT technique. In this study, the possibility of center-specific bias was excluded because patients were recruited through Taiwan on the basis of the TCRD. Furthermore, the health insurance program in Taiwan covers positron emission tomography (PET) and an endoscopic ultrasound for every patient with TESCC. In our study, all patients with TESCC were staged using PET and an endoscopic ultrasound, which was confirmed on the basis of the medical procedure codes of PET and endoscopic ultrasound codes in the insurance database.

In Taiwan, all radiation oncologists have been well trained, re-educated, and passed the Board Certification of Taiwan Society for Therapeutic Radiology and Oncology. The standard protocols of quality control for the contouring or those in the RT manual for TESCC were based on the consensus contouring guidelines of the Association of Residents in Radiation Oncology, and the dose constraints to organs at risk were as per the NCCN guidelines for the RT of esophageal cancer [7,34]. Additionally, all treatment guidelines that include chemotherapy regimens, total irradiation dose, fraction size, and treatment plans of RT must be surveyed and verified by Taiwan’s General Cancer Certification Program administered by the Bureau of Health Promotion every three-years [35]. If the criteria for the General Cancer Certification Program are not fulfilled, patients with cancer cannot receive cancer treatments in the hospital [35]. Quality control for the delivery of fractions in various medical centers across Taiwan was satisfactory.

Comorbidities were scored using the Charlson comorbidity index (CCI) [36,37]. Only comorbidities observed in the six-months before the index date were included, and these were coded and classified according to the International Classification of Diseases, Ninth Revision, Clinical Modification (ICD-9-CM) codes at the first admission or after more than two repetitions of a code were issued at outpatient department visits. Significant independent predictors, namely, radiation modalities, age, sex, CCI score, AJCC clinical stage, and diagnosis year, were analyzed using multivariate Cox regression analysis to determine hazard ratios (HRs). Analysis was adjusted for, and stratified by, independent predictors. The endpoint was the mortality rate among treatments, and Group 1 was used as the control. The toxicity profile after CCRT was also evaluated, and on the basis of major heart events, toxicity was defined as a diagnosis of myocardial infarction, coronary revascularization, or death from ischemic heart disease [38]; patients with TESCC requiring narcotic antitussives, oxygen, steroids, or assisted ventilation after CCRT were considered to have radiation pneumonitis at grade 2 or higher [39].

The cumulative incidence of death was estimated using the Kaplan–Meier method, and differences between 3D-CRT-based CCRT and IMRT-based CCRT were determined using the log-rank test. After adjustment for confounders, the Cox proportional method was used to determine the time from the index date to all-cause mortality in patients who received IMRT-based CCRT or 3D-CRT-based CCRT. Subsequently, in multivariate analysis, HRs were adjusted for age, sex, CCI score, AJCC clinical stage, diagnosis year, and RT modalities. Stratified analyses were conducted to evaluate the mortality risk associated with different RT modalities and with AJCC clinical stages. All analyses were conducted using SAS (Version 9.3; SAS, Cary, NC, USA), and a two-tailed *p* value of <0.05 was considered statistically significant.

## 3. Results

We enrolled 2062 patients with TESCC who had received CCRT and categorized them into two groups, on the basis of their treatment modality, to compare treatment outcomes: Group 1 (received 3D-CRT-based CCRT) and Group 2 (received IMRT-based CCRT). The median total dose and fraction size of RT in Groups 1 and 2 were both 50.40 Gy. Among these patients, 538 received 3D-CRT-based CCRT (Group 1), and 1524 received IMRT-based CCRT (Group 2). The dosage distribution of irradiation and chemotherapy between Groups 1 and 2 was homogenous; the median irradiation dose in Groups 1 and 2 was 50.40 Gy. The median follow-up duration after the index date was 2.02 years. Groups 1 and 2 did not differ significantly in age, follow-up duration, sex, CCI score, toxicity profile, irradiation dose, or dosage of chemotherapy (Table 1). However, the proportion of patients with advanced stage TESCC in Group 1 (80.67%) was lower than that in Group 2 (84.78%), and the mortality rate was 84.76% and 75.59% in Groups 1 and 2, respectively. From 2011 to 2014, 70.47% and 35.32% of patients with TESCC received IMRT-based CCRT and 3D-CRT-based CCRT, respectively, whereas from 2006 to 2011, 29.53% and 64.68% of patients with TESCC received IMRT-based CCRT and 3D-CRT-based CCRT, respectively. 

Multivariate Cox regression analysis indicated that advanced AJCC clinical stages (≥IIIA) and 3D-CRT were significant and poor independent predictors in patients with TESCC who received definitive CCRT (Table 2). Moreover, IMRT-based CCRT (adjusted HR [aHR]: 0.88, 95% confidence interval [CI]: 0.78–0.98) was a significant independent prognostic factor for overall survival (*p* = 0.0223; Table 2).

The AJCC clinical stage was a crucial independent predictor of overall survival. Furthermore, increased aHRs were found for advanced stages (aHR: 1.89, 95% CI: 1.63–2.19; Table 2). Table 3 presents the results of the stratified analysis of mortality risk associated with 3D-CRT-based CCRT and IMRT-based CCRT and with different AJCC clinical stages. To investigate post-treatment mortality risk, Group 1 (standard CCRT dose with 3D-CRT: Median, 50.40 Gy) was used as the control. After adjustment for age, sex, CCI score, clinical AJCC stage, toxicity profile, and diagnosis year, aHRs (95% CIs) for the overall mortality at all clinical stages were 0.88 (0.78–0.98, *p* = 0.0223) in Group 2. By contrast, aHRs (95% CIs) for the overall mortality at early (IA–IIB) and advanced clinical stages were 0.91 (0.67–1.25, *p* = 0.5746) and 0.88 (0.77–0.99, *p* = 0.0368), respectively, in Group 2 (Table 3). 

The clinical T and N stages were also crucial independent predictors of overall survival. The T and N stages of the IMRT and 3D-CRT groups are presented in Supplementary Table S1. Furthermore, increased aHRs were found for the advanced T3 and T4 stages (aHR: 1.19, 95% CI: 1.04–1.25 and aHR: 1.19, 95% CI: 1.04–1.25 for T2 and T3, respectively; Supplementary Table S2). Increased aHRs were found for advanced N1, N2, and N3 stages (aHR: 1.12, 95% CI: 1.09–1.21, aHR: 1.39, 95% CI: 1.29–2.08, and aHR: 1.99, 95% CI: 1.87–2.43, respectively; Supplementary Table S2).

Supplementary Table S3 presents the results of the stratified analysis of mortality risk associated with 3D-CRT-based CCRT and IMRT-based CCRT and with different clinical T and N stages. After adjustment for age, sex, CCI score, clinical T stages, clinical N stages, toxicity profile, and diagnosis year, aHRs (95% CIs) for the overall mortality at all clinical stages were 0.86 (0.75–0.91, *p* = 0.0116) in Group 2. By contrast, aHRs (95% CIs) for the overall mortality at early T and advanced T stages were 0.98 (0.77–2.25, *p* = 0.9854) and 0.86 (0.74–0.96, *p* = 0.0277) in Group 2, respectively (Supplementary Table S3). aHRs (95% CIs) for the overall mortality at early N and advanced N stages were 0.91 (0.67–1.95, *p* = 0.5746) and 0.83 (0.74–0.94, *p* = 0.0140), respectively, in Group 2 (Supplementary Table S3).

We applied propensity score matching (PSM) to reduce the effects of confounders. The confounders were RT modalities, sex, age, CCI score, year of diagnosis, and AJCC clinical stages. Through PSM executed using the global optimum method [40], we matched patients in 3D-CRT with those in the remaining groups at a 1:1 ratio (Supplementary Table S4). We applied the Cox proportional hazards model to evaluate the cumulative incidence of death in patients in various groups (Supplementary Table S5). The trend suggested that IMRT was still superior to 3D-CRT.

**Figure 1:**
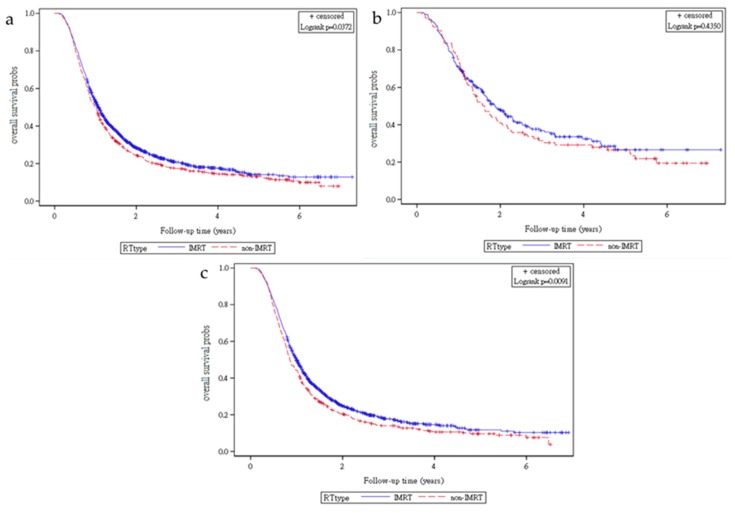
Kaplan–Meier curves for overall survival in patients undergoing various treatments. (**a**) All AJCC clinical stages. (**b**) Early AJCC clinical stage IA-IIB. (**c**) Advanced AJCC clinical stage IIIA-IIIB.

Figure 1 presents Kaplan–Meier curves that illustrate the overall survival of patients in both treatment groups. Specifically, two-year overall survival rates at all clinical stages were 34.66% and 28.89% in Groups 2 and 1, respectively (Figure 1a); however, the overall survival rate was higher in Group 2 (log-rank test, *p* = 0.0372) than in Group 1. Additionally, two-year overall survival rates in patients with early-stage TESCC were 49.14% and 39.77%, respectively, in Groups 2 and 1 (Figure 1b; log-rank test, *p* = 0.4350). By contrast, two-year overall survival rates among patients with advanced stage TESCC were 27.73% and 20.98% in Groups 2 and 1, respectively (Figure 1c; log-rank test, *p* = 0.0091).

## 4. Discussion

More than 60% of patients with esophageal cancer in the INT 0123 trial were at early clinical stages [8]. The RT field included relatively small tumor sizes and a less extensive lymph node field in the INT 0123 trial compared with the RT field in stages IIIA and IIIB [26]. CCRT with conventional RT techniques in a relatively small RT field in the INT 0123 trial might be feasible because >60% of patients were at early stages; however, CCRT with the conventional RT technique might be more toxic to Taiwanese patients with TESCC (>80% of patients with TESCC were at stage IIIA–IIIC) than IMRT-based CCRT [3,4,22,23]. For the TESCC treatment, contemporary RT techniques with IMRT might provide greater tumor coverage or fewer iatrogenic complications, as well as higher overall survival rates [4,19,22,40].

This study is the first to estimate the mortality rates of patients with TESCC who received IMRT-CRT-based or 3D-CRT-based CCRT, as per the standard CCRT protocol from the INT 0123 trial and based on NCCN guidelines. Mixed adenocarcinoma and SCC were also analyzed in the INT 0123 trial, and patients in the trial had cervical and thoracic esophageal cancer as well as GE cancer, which might result in varying survival rates and different optimal treatment options compared with patients with TESCC who received definitive CCRT [41,42,43,44,45].

Scatter irradiation dose–volume to the heart or lung has been reported to be lower at the cervical or GE-junction portions of esophageal cancer than at the thoracic portion [24,27,28]. For TESCC patients at advanced clinical stages, conventional RT, namely the CCRT protocol of the INT 0123 trial, might be relatively toxic to the lungs or heart and may provide less coverage to the primary tumor and regional lymph nodes because larger RT fields are used in advanced stages [26]. Modern RT techniques that are based on the INT 0123 protocol and NCCN guidelines might improve patient outcomes and reduce the toxicity of CCRT to patients with TESCC, which would result in low radiation-related toxicity and better tumor control in such patients [21,22,40]. Heterogenous inclusion criteria including pathological types, tumor locations, clinical stages, and conventional RT techniques may thus be inadequate to assess the survival of patients with TESCC receiving IMRT because TESCC tends to recur locoregionally first, whereas GE cancer and esophageal adenocarcinoma more commonly recur with distant dissemination [11,12,13,14,15]. IMRT, which has greater tumor coverage and a higher conformity index compared with 3D-CRT, might result in a higher local control rate; its superior local control rate may translate to survival benefits in patients with TESCC who have a high risk of local recurrence [19,21,22]. Therefore, in this study, early and advanced clinical stage-stratified analysis was conducted to clarify the effects of IMRT in patients with TESCC; notably, this study is the first to report that IMRT-based CCRT is a superior therapy for patients who received 3D-CRT, particularly in those at advanced stages (Table 2, Table 3, and Figure 1).

According to our review of the literature, this study is the leading and largest study to estimate the effect of IMRT or 3D-CRT on overall survival in patients with TESCC. New radiation techniques such as IMRT are associated with more favorable toxicity profiles [22,23]. IMRT is superior to conventional RT techniques in terms of conformity and dose homogeneity to the target [46,47,48,49,50]. Most studies have reported that IMRT can spare the lungs more effectively with respect to the lung volume that would receive >20.00 Gy [46,47,50], the mean lung dose (MLD) [48,49], and the lung volume receiving >10.00 Gy or >5.00 Gy [47]. Many studies have reported that lung volumes that receive >20.00 Gy and MLD are prone to radiation pneumonitis [51,52,53,54]. IMRT can significantly improve lung sparing in terms of the MLD and lung volume receiving >20.00 Gy; theoretically, IMRT could reduce pulmonary complications [53,54,55,56]. Some studies have reported that IMRT can reduce the radiation dose to the heart in terms of heart volume exposed to >30.00 Gy, 40.00 Gy, mean heart dose, and the right coronary artery [50,55,57]. Thus, IMRT has a considerably greater potential to achieve a higher degree of target conformity and sparing of normal tissue than conventional RT techniques, particularly for target volumes or organs at risk of complex shapes or concave regions. However, whether the dosimetry advantage of IMRT translates into better clinical outcomes remains unclear. 

The previous, largest series was conducted by Lin et al. in the United States; comparing long-term outcomes between 3D-CRT and IMRT, overall survival rates were significantly higher, locoregional control was significantly higher, and cardiac death was significantly lower after IMRT than after 3D-CRT [40]. Lin et al. demonstrated that more than 80% of patients with esophageal cancer had malignancies in the lower portion of the esophagus [40]. Notably, patients with adenocarcinoma or SCC were included in their study [40]. In the study by Lin et al., patients with esophageal cancer received sequential chemotherapy and RT instead of standard definitive CCRT; additionally, neoadjuvant chemotherapy was administered as combinations of 5-fluorouracil and taxane or with platinum-based compounds instead of the cisplatin-based compounds used in standard CCRT [8]. More than 45% of patients in the study by Lin et al. received surgery after RT; hence, the survival benefits related to IMRT, surgery, neoadjuvant chemotherapy, different esophageal portions, different chemotherapy regimens, or different pathological types remain unclear [40]. Insufficient extrapolation of the survival benefits of IMRT has been applied in daily current practice on the basis of the NCCN guidelines [7]. 

By contrast, our study had a longer follow-up time, a large group of patients with TESCC, and homogenous regimens and dosages of irradiation and chemotherapy (Table 1). Compared with the study by Lin et al., our study is novel because we demonstrated which patients were optimal candidates for IMRT among patients with TESCC at advanced clinical stages (Table 3). We conducted a leading large cohort study to compare the treatment outcomes of IMRT-CRT-based or 3D-CRT-based CCRT for TESCC. Our findings suggest that, compared with 3D-CRT, standard radiation doses of IMRT improve overall survival in patients with advanced stages (IIIA-IIIC) of TESCC (Table 3 and Figure 1).

In Table 1, we did not use propensity score matching because most covariates were similar. A higher proportion of advanced clinical stages, higher use of IMRT from 2011 to 2014, and a higher crude survival rate in the IMRT group were observed. The number of advanced stages was higher in the IMRT group than in the 3D-CRT group. The death rate was lower in the IMRT group than in the 3D-CRT group after Cox multivariate analysis (Table 2). Thus, the benefits of an increased survival rate, engendered by IMRT, could only be underestimated; hence, the conclusions of this study should remain valid. 

From 2011 to 2014, 70% of patients with TESCC received IMRT. In the past 10 years, no change in the specific therapeutic policies of TESCC occurred; definitive CCRT, following the regimens of INT0123, has been the standard treatment for TESCC if patients did not receive surgery. The one improvement is that the RT technique changed from 3D-CRT to IMRT. However, the question of whether progression of irradiation dosimetry improves survival in TESCC remains unanswered.

We attempted to estimate the survival effects of IMRT in patients with TESCC who received standard CCRT regimens. Local control is more crucial in ESCC than in esophageal adenocarcinoma because of a higher risk of local recurrence in esophageal adenocarcinoma than in ESCC [11,12,13,14,15]. According to our other studies [2,3,4], patients with esophageal adenocarcinomas have poor overall survival because most patients die of distant metastasis compared with ESCC patients [13,14]. ESCC patients have better overall survival, but more have a failure at locoregional sites compared with esophageal adenocarcinoma patients [11,12,13,14,15]. Therefore, local control is essential in TESCC to decrease local recurrence and to prolong overall survival [11,12,13,14,15].

In Taiwan, palliative systemic therapy (e.g., chemotherapy or immune therapy) or the best practices of supportive care for TESCC with local recurrence are the main treatments based on the NCCN guidelines [7], because previous irradiation increases surgical complications and mortality [58]. Once patients with TESCC have local recurrence after CCRT, survival rates are dismal [59]. Compared with 3D-CRT, IMRT has been reported to have better dosimetry for tumor control and lower irradiation scatter dose to the lungs and heart, which may be the reasons for higher survival after IMRT [21,22,40].

Cox proportional hazard regression analysis of the risk of death among patients with TESCC receiving CCRT demonstrated that the mortality rate was lower in the IMRT group than in the 3D-CRT group (Table 2). AJCC clinical stages were also independent prognostic factors for the mortality rate. The finding that the AJCC clinical stage was an independent prognostic factor in patients with TESCC receiving CCRT was compatible with those of other studies [60,61]. After stratified analysis of clinical stages, IMRT remained an independent prognostic factor for low mortality risk in patients with TESCC at all stages, and advanced stages were treated on the basis of standard CCRT regimens (Table 3, Supplementary Table 3, Figure 1a, and Figure 1c). No statistically significant change in early clinical stages (IA–IIB) was observed. The findings might be associated with a relatively small primary tumor and the narrow field of lymph node regions; furthermore, 3D-CRT could cover early clinical stage primary tumors well, and relatively low scatter radiation doses were incidental in the lungs and heart. Hence, the advantages of IMRT were not obvious in patients with early stages of TESCC (Figure 1b). 

Our results suggest that standard dose IMRT-based CCRT is more beneficial than 3D-CRT in overall survival in patients with advanced stages (IIIA-IIIC) of TESCC. For early clinical stages (IA-IIB), regardless of the standard dose, IMRT-based CCRT or 3D-CRT-based CCRT might be a feasible and favorable choice. According to our review of the literature, no peer-reviewed or large cohort study on patients with TESCC at different stages receiving the IMRT techniques in standard CCRT regimens have been conducted. This study is the first to compare IMRT-based CCRT with 3D-CRT-based CCRT. Compared with the 3D-CRT-based CCRT, IMRT-based CCRT resulted in higher survival rates among patients with advanced clinical stages (IIIA-IIIC) of TESCC. Thus, IMRT should be the standard RT technique in definitive CCRT for patients with TESCC. 

This study had limitations. First, the toxicity induced by the treatments was not determined; therefore, treatment-related mortality estimates may be biased. Patients might die of cancer earlier compared with deaths caused by treatment-related toxicity. Competing risks might occur in our analysis when a subject is at risk of more than one type of event [62]. Second, because all patients with TESCC were enrolled from an Asian population, the corresponding ethnic susceptibility remains unclear; hence, our results should be cautiously extrapolated to non-Asian populations. Third, the stratification of a relatively low number of patients with early stages of TESCC receiving CCRT after staging might limit the generalizability of our conclusions. The trend of survival benefits, in the early stages for patients with TESCC and undergoing IMRT-based CCRT, might be statically significant with a larger sample. 

Fourth, diagnoses of all comorbidities were completely dependent on ICD-9-CM codes. Nevertheless, the Taiwan Cancer Registry Administration randomly reviewed charts and interviewed patients to verify the accuracy of the diagnoses, and hospitals with outlier chargers or practices could undergo an audit and subsequently receive costly penalties if malpractice or discrepancies were identified. Fifth, selection bias might have occurred because IMRT-based CCRT was the treatment for relatively large and unresectable TESCC with the same clinical stages. However, we might have underestimated the effect of IMRT-based CCRT. Therefore, to obtain crucial information concerning population specificity and disease occurrence, a large-scale randomized trial comparing carefully selected patients undergoing suitable treatments is essential. Sixth, we used an overall follow-up duration of two-years. However, the median survival time of all stages of patients with TESCC in Taiwan receiving CCRT was 293 days (9.77 months) [61]; thus, two-year (24 months) follow-ups were sufficient for high early mortality patients with cancer, especially in advanced stages. In addition, Kaplan–Meier curves for overall survival never intersected before the fifth year, especially in advanced stages (Figure 1a,c); the sample size was large; and >80% of mortality was observed in patients with TESCC at the five-year follow-up. Therefore, using a longer follow-up would not overturn the conclusion. 

Finally, the Cancer Registry Database did not contain information regarding dietary habits, socioeconomic status, or body mass index, and all these factors may be risk factors for mortality. However, considering the magnitude and statistical significance of the observed effects in this study, these limitations are unlikely to affect the conclusions.

## 5. Conclusions

IMRT-based CCRT resulted in higher survival rates in patients with advanced IIIA–IIIC stages, clinical T3, clinical T4 stages, or lymph node involvement of TESCC compared with 3D-CRT-based CCRT.

## Figures and Tables

**Table 1:** Characteristics of patients with thoracic esophageal squamous cell carcinoma (TESCC) receiving concurrent chemoradiotherapy (CCRT) with intensity-modulated radiation therapy (IMRT) or 3D-CRT (conformal radiation therapy).

Variable	All		IMRT		3D-CRT		
	*n* = 2062	(%)	*n* = 1524	(%)	*n* = 538	(%)	*p* Value
**Age**							
Mean ± SD	57.92	±11.00	57.85	±10.86	58.44	±11.37	0.1342
							0.5096
20–29	6	(0.29)	4	(0.26)	2	(0.37)	
30–39	55	(2.67)	40	(2.62)	15	(2.79)	
40–49	472	(23.09)	345	(22.64)	127	(23.61)	
50–59	714	(34.82)	545	(35.76)	169	(31.41)	
60–69	500	(23.97)	367	(24.08)	133	(24.72)	
70–79	240	(11.45)	168	(11.02)	72	(13.38)	
80–89	72	(3.52)	52	(3.41)	20	(3.72)	
90–100	3	(0.14)	3	(0.20)	0	(0.00)	
**Sex**							0.4189
Male	1960	(95.05)	1447	(94.95)	513	(95.35)	
Female	102	(4.95)	77	(5.05)	25	(4.65)	
**Follow-up time (year)**							0.1848
Median, IQR	2.02	1.15	2.02	1.15	2.00	1.14	
(q1, q3)	(0.92,	2.77)	(0.93,	2.78)	(0.99,	2.74)	
(min, max)	(0.09,	7.29)	(0.09,	7.29)	(0.12,	6.96)	
**CCI score**							
mean ± SD	1.25	±1.39	1.25	±1.40	1.29	±1.34	0.4568
0	697	(34.03)	533	(34.97)	164	(30.48)	0.2443
1	721	(35.10)	525	(34.45)	196	(36.43)	
2	353	(16.78)	253	(16.60)	100	(18.59)	
>3	291	(14.09)	213	(13.98)	78	(14.50)	
**Radiation dose (Gy)**							0.9617
Median, IQR	50.40	±0.30	50.40	±0.31	50.40	±0.22	
**Cumulative cisplatin dose** **(mg/m^2^)**							0.1152
Mean (SD)	255.57	±57.65	255.57	±58.61	253.56	±59.16	
**AJCC clinical stages**							0.0017
Early stage (IA-IIB)	336	(16.78)	232	(15.22)	104	(19.33)	
Advanced stage (IIIA-IIIC)	1726	(83.22)	1292	(84.78)	434	(80.67)	
**AJCC clinical stages**							<0.0001
IA	5	(0.42)	5	(0.33)	0	(0.00)	
IB	31	(1.62)	24	(1.57)	7	(1.30)	
IIA	126	(6.31)	79	(5.18)	47	(8.74)	
IIB	174	(8.44)	124	(8.14)	50	(9.29)	
IIIA	397	(19.19)	319	(20.93)	78	(14.50)	
IIIB	661	(31.80)	439	(28.81)	219	(40.71)	
IIIC	671	(32.22)	534	(35.04)	137	(25.46)	
**Year of diagnosis**							<0.0001
2006–2010	798	(39.08)	450	(29.53)	348	(64.68)	
2011–2014	1264	(60.92)	1074	(70.47)	190	(35.32)	
**Toxicity profile**							0.5687
Major heart events	58	(2.81)	41	(2.69)	17	(3.16)	
Radiation pneumonitis grade 2	465	(22.55	334	(21.92)	122	(22.67)	
**Death**							<0.0001
No	454	(21.84)	372	(24.41)	82	(15.24)	
Yes	1608	(78.16)	1152	(75.59)	456	(84.76)	

CCRT, concurrent chemoradiotherapy; CCI, Charlson comorbidity index; RT, radiotherapy; CCRT, concurrent chemoradiotherapy; AJCC, American Joint Committee on Cancer; IMRT, intensity-modulated radiation therapy; IQR, interquartile range; SD, standard deviation; Gy, Gray; 3D-CRT, three-dimensional conformal radiation therapy.

**Table 2:** Cox proportional hazard regression analysis of mortality risk among patients with TESCC receiving concurrent chemoradiotherapy (CCRT).

Variable	Univariate Analysis				Multivariate Analysis			
	HR	95% CI		*p*	aHR *	95% CI		*p*
**RT Modalities**								
3D-CRT (ref)	1				1			
IMRT	0.89	(0.80,	0.99)	0.0372	0.88	(0.78,	0.98)	0.0223
**Sex**								
Female (ref)	1				1			
Male	1.12	(0.89,	1.40)	0.3378	1.06	(0.84,	1.33)	0.6274
**Age**								
20–29 (ref)	1				1			
30–39	2.07	(0.29,	14.86)	0.4680	2.30	(0.32,	16.71)	0.4098
40–49	2.25	(0.32,	15.88)	0.4149	2.44	(0.34,	17.42)	0.3735
50–59	1.79	(0.25,	12.59)	0.5600	1.98	(0.28,	14.12)	0.4958
60–69	1.65	(0.23,	11.65)	0.6141	1.78	(0.25,	12.72)	0.5649
70–79	1.62	(0.23,	11.42)	0.6311	1.89	(0.26,	13.57)	0.5251
80–89	1.92	(0.27,	13.76)	0.5144	2.17	(0.30,	15.73)	0.4433
90–	2.14	(0.23,	20.44)	0.5075	3.09	(0.32,	29.79)	0.3295
**CCI score**								
0 (ref)	1				1			
1	0.98	(0.87,	1.11)	0.7628	1.02	(0.91,	1.15)	0.7646
2	0.94	(0.81,	1.08)	0.3725	1.02	(0.88,	1.18)	0.7948
>3	0.96	(0.83,	1.12)	0.6291	1.14	(0.97,	1.33)	0.1176
**Year of diagnosis**								
2006–2010 (ref)	1				1			
2011–2014	0.96	(0.87,	1.07)	0.4678	0.96	(0.87,	1.07)	0.4741
**AJCC clinical stages**								
Early stage (ref)	1				1			
Advanced stage	1.84	(1.59,	2.12)	<0.0001	1.89	(1.63,	2.19)	<.0001

* All variables were used in multivariate analysis. HR, hazard ratio; CI, confidence interval; aHR, adjusted hazard ratio. CCRT, concurrent chemoradiotherapy; CCI, Charlson comorbidity index; RT, radiotherapy; CCRT, concurrent chemoradiotherapy; AJCC, American Joint Committee on Cancer; ref, reference group; IMRT, intensity-modulated radiation therapy; 3D-CRT, three-dimensional conformal radiation therapy.

**Table 3:** American Joint Committee on Cancer (AJCC)-stage-stratified Cox proportional hazards model for mortality risk associated with treatment modalities in patients with TESCC receiving concurrent chemoradiotherapy (CCRT).

Treatment	*n*	Death	Death Rate (%)	Univariate Analysis				Multivariate Analysis			
				HR	95% CI		*p* Value	aHR *	95% CI		*p* Value
**All stage**	2062	1608									
3D-CRT	538	456	84.76	1				1			
IMRT	1524	1152	75.59	0.89	(0.80,	0.99)	0.0372	0.88	(0.78,	0.98)	0.0223
**Early stage (IA~IIB)**	336	218									
3D-CRT	104	77	74.04	1				1			
IMRT	232	141	60.78	0.89	(0.68,	1.18)	0.4344	0.91	(0.67,	1.25)	0.5746
**Advanced stage (IIIA-IIIC)**	1726	1390									
3D-CRT	434	379	87.33	1				1			
IMRT	1292	1011	78.25	0.85	(0.76,	0.96)	0.0091	0.88	(0.77,	0.99)	0.0368

* All variables in Table 2 were used in multivariate analysis. HR, hazard ratio; CI, confidence interval; aHR, adjusted hazard ratio. CCRT, concurrent chemoradiotherapy; CCI, Charlson comorbidity index; RT, radiotherapy; CCRT, concurrent chemoradiotherapy; AJCC, American Joint Committee on Cancer; ref, reference group; IMRT, intensity-modulated radiation therapy; 3D-CRT, three-dimensional conformal radiation therapy.

## Supplementary Materials

The following are available online at https://www.mdpi.com/2072-6694/11/10/1529/s1, Table S1: Characteristics of patients with TESCC receiving CCRT with intensity-modulated radiation therapy or three-dimensional conformal radiation therapy (AJCC clinical stages presented with TN system), Table S2: Cox proportional hazard regression analysis of mortality risk among patients with TESCC receiving CCRT (AJCC clinical stages presented with TN system), Table S3: T and N-stage-stratified Cox proportional hazards model for mortality risk associated with treatment modalities in patients with TESCC receiving CCRT. Table S4: Characteristics of patients with TESCC receiving CCRT with IMRT or 3D-CRT and their propensity score–matched cohort. Table S5: Cox proportional hazard regression model for mortality risk among patients with TESCC receiving CCRT in their propensity score–matched cohort.

## Abbreviations Lis

RT	radiotherapy
IMRT	intensity-modulated radiotherapy
ICD-9-CM	International Classification of Diseases, Ninth Revision, Clinical Modification
CCRT	concurrent chemoradiotherapy
AJCC	American Joint Committee on Cancer
HR	hazard ratio
CI	confidence interval
CCI	Charlson comorbidity index
TESCC	thoracic esophageal squamous cell carcinoma
ESCC	esophageal squamous cell carcinoma
SCC	squamous cell carcinoma
3D-CRT	three-dimensional conformal radiation therapy
NCCN	National Comprehensive Cancer Network
TCRD	Taiwan Cancer Registry Database
