# Supplementary material for: Three-Dimensional Conformal Radiotherapy-Based or Intensity-Modulated Radiotherapy-Based Concurrent Chemoradiotherapy in Patients with Thoracic Esophageal Squamous Cell Carcinoma
Source: Cancers (Basel). 2019 Oct 10;11(10):1529. doi: 10.3390/cancers11101529 (PMC6826542; doi:10.3390/cancers11101529)
Supplement: Supplementary file 1 [file cancers-11-01529-s001.pdf]

Supplementary Marterials: Three-Dimensional Conformal Radiotherapy-Based or Intensity-Modulated Radiotherapy-Based Concurrent Chemoradiotherapy in Patients with Thoracic Esophageal Squamous Cell Carcinoma

Wei-Cheng Lin, Chia-Lun Chang, Han-Lin Hsu, Kevin Sheng-Po Yuan, Alexander T.H. Wu and Szu-Yuan Wu

Table S1. Characteristics of patients with TESCC receiving CCRT with intensity-modulated radiation therapy or three-dimensional conformal radiation therapy (AJCC clinical stages presented with TN system).

Variables	All		IMRT		3D-CRT		*P* value
	n = 2062	(%)	n = 1524	(%)	n = 538	(%)	
**Age**							
Mean ± SD	57.92	± 11.00	57.85	± 10.86	58.44	± 11.37	0.1342
							0.5096
20-29	6	(0.29)	4	(0.26)	2	(0.37)	
30-39	55	(2.67)	40	(2.62)	15	(2.79)	
40-49	472	(23.09)	345	(22.64)	127	(23.61)	
50-59	714	(34.82)	545	(35.76)	169	(31.41)	
60-69	500	(23.97)	367	(24.08)	133	(24.72)	
70-79	240	(11.45)	168	(11.02)	72	(13.38)	
80-89	72	(3.52)	52	(3.41)	20	(3.72)	
90-100	3	(0.14)	3	(0.20)	0	(0.00)	
**Sex**							0.4189
Male	1960	(95.05)	1447	(94.95)	513	(95.35)	
Female	102	(4.95)	77	(5.05)	25	(4.65)	
**Follow-up time(year)**							0.1848
Median, IQR	2.02	1.15	2.02	1.15	2.00	1.14	
(q1, q3)	(0.92,	2.77)	(0.93,	2.78)	(0.99,	2.74)	
(min, max)	(0.09,	7.29)	(0.09,	7.29)	(0.12,	6.96)	
**CCI score**							
mean ± SD	1.25	± 1.39	1.25	± 1.40	1.29	± 1.34	0.4568
0	697	(34.03)	533	(34.97)	164	(30.48)	0.2443
1	721	(35.10)	525	(34.45)	196	(36.43)	
2	353	(16.78)	253	(16.60)	100	(18.59)	
>3	291	(14.09)	213	(13.98)	78	(14.50)	
**Radiation dose (Gy)**							0.9617
Median, IQR	50.40	± 0.30	50.40	± 0.31	50.40	± 0.22	
**Cumulative cisplatin dose (mg/m^2)**							0.1152
Mean (SD)	255.57	± 57.65	255.57	± 58.61	253.56	± 59.16	
**Clinical T stages**							<0.0001
T1	5	(0.42)	5	(0.33)	0	(0.00)	
T2	131	(6.35)	66	(4.33)	65	(7.43)	
T3	816	(39.57)	549	(36.02)	267	(38.48)	
T4	1110	(53.83)	904	(59.31)	206	(38.28)	
**Clinical N stages**							<0.0001
N0	316	(15.32)	218	(14.30)	98	(18.22)	
N1	401	(19.44)	274	(17.98)	127	(23.61)	
N2	721	(34.97)	543	(35.63)	178	(33.09)	
N3	624	(30.26)	489	(32.09)	135	(25.09)	
**Year of diagnosis**							<0.0001
2006-2010	798	(39.08)	450	(29.53)	348	(64.68)	
2011-2014	1264	(60.92)	1074	(70.47)	190	(35.32)	
**Toxicity profile**							0.5687
Major heart events	58	(2.81)	41	(2.69)	17	(3.16)	

Radiation pneumonitis grade 2	465	(22.55)	334	(21.92)	122	(22.67)	
**Death**							<0.0001
No	454	(21.84)	372	(24.41)	82	(15.24)	
Yes	1608	(78.16)	1152	(75.59)	456	(84.76)	

CCRT, concurrent chemoradiotherapy; CCI, Charlson comorbidity index; RT, radiotherapy; CCRT, concurrent chemoradiotherapy; AJCC, American Joint Committee on Cancer; ref, reference; IMRT, intensity-modulated radiation therapy; IQR, interquartile range; SD, standard deviation; Gy, Gray; 3D-CRT, three-dimensional conformal radiation therapy.

Table S2. Cox proportional hazard regression analysis of mortality risk among patients with TESCC receiving CCRT (AJCC clinical stages presented with TN system).

Variable	Univariate analysis				Multivariate analysis			
	HR	95% CI		*P*	aHR*	95% CI		*P*
**RT Modalities**								
3D-CRT (ref)	1				1			
IMRT	0.89	(0.80,	0.99)	0.0372	0.86	(0.75,	0.91)	0.0116
**Sex**								
Female (ref)	1				1			
Male	1.12	(0.89,	1.40)	0.3378	1.04	(0.91,	1.36)	0.5729
**Age**								
20-29 (ref)	1				1			
30-39	2.07	(0.29,	14.86)	0.4680	2.24	(0.41,	16.59)	0.3987
40-49	2.25	(0.32,	15.88)	0.4149	2.33	(0.37,	16.75)	0.4613
50-59	1.79	(0.25,	12.59)	0.5600	1.74	(0.34,	13.91)	0.4847
60-69	1.65	(0.23,	11.65)	0.6141	1.75	(0.28,	11.61)	0.6538
70-79	1.62	(0.23,	11.42)	0.6311	1.87	(0.25,	13.87)	0.6140
80-89	1.92	(0.27,	13.76)	0.5144	2.15	(0.35,	13.40)	0.5215
90-	2.14	(0.23,	20.44)	0.5075	3.01	(0.52,	27.61)	0.4184
**CCI score**								
0 (ref)	1				1			
1	0.98	(0.87,	1.11)	0.7628	1.03	(0.92,	1.11)	0.8313
2	0.94	(0.81,	1.08)	0.3725	1.01	(0.89,	1.08)	0.8927
>3	0.96	(0.83,	1.12)	0.6291	1.09	(0.96,	1.32)	0.1267
**Year of diagnosis**								
2006~2010 (ref)	1				1			
2011~2014	0.96	(0.87,	1.07)	0.4678	0.97	(0.89,	1.09)	0.5830
**Clinical T stages**								
T1 (ref)	1				1			
T2	1.03	(0.77,	1.13)	0.8537	1.02	(0.94,	1.12)	0.8735
T3	1.23	(1.08,	1.15)	0.0231	1.19	(1.04,	1.25)	0.0345
T4	1.96	(1.35,	2.43)	<.0001	1.94	(1.34,	2.23)	<0.0001
**Clinical N stages**								
N0 (ref)	1				1			
N1	1.08	(1.04,	1.23)	0.0342	1.12	(1.09,	1.21)	0.0342
N2	1.41	(1.27,	1.97)	<.0001	1.39	(1.29,	2.08)	<0.0001
N3	2.16	(1.71,	2.26)	<.0001	1.99	(1.87,	2.43)	<0.0001

*All variables were used in multivariate analysis. HR, hazard ratio; CI, confidence interval; aHR, adjusted hazard ratio. CCRT, concurrent chemoradiotherapy; CCI, Charlson comorbidity index; RT, radiotherapy; CCRT, concurrent chemoradiotherapy; AJCC, American Joint Committee on Cancer; ref, reference group; IMRT, intensity-modulated radiation therapy; 3D-CRT, three-dimensional conformal radiation therapy.

Table S3. T and N-stage-stratified Cox proportional hazards model for mortality risk associated with treatment modalities in patients with TESCC receiving CCRT.

Treatment				Univariate analysis				Multivariate analysis			
	n	death	death rate (%)	HR	95% CI		P value	aHR*	95% CI		P value
**All stage**	2062	1608									
3D-CRT	538	456	84.76	1				1			
IMRT	1524	1152	75.59	0.89	(0.80,	0.99)	0.0372	0.86	(0.75,	0.91)	0.0116
**Early T stage (T1-T2)**	136	81									
3D-CRT	65	39	60.00	1				1			
IMRT	71	42	59.15	0.99	(0.71,	2.32)	0.8677	0.98	(0.77,	2.25)	0.9854
**Advanced T stage (T3-T4)**	1926	1527									

3D-CRT	473	402	84.99	1				1			
IMRT	1453	1125	77.43	0.84	(0.74,	0.91)	0.0037	0.86	(0.74,	0.96)	0.0277
**Early N stage (N0)**	316	218									
3D-CRT	98	68	69.39	1				1			
IMRT	218	150	68.81	0.89	(0.68,	1.98)	0.4344	0.91	(0.67,	1.95)	0.5746
**Advanced N stage (N1-N3)**	1746	1390									
3D-CRT	440	391	88.86	1				1			
IMRT	1306	999	76.49	0.81	(0.71,	0.92)	0.0015	0.83	(0.74,	0.94)	0.0140

*All variables in Supplemental Table 2 were used in multivariate analysis. HR, hazard ratio; CI, confidence interval; aHR, adjusted hazard ratio. CCRT, concurrent chemoradiotherapy; CCI, Charlson comorbidity index; RT, radiotherapy; CCRT, concurrent chemoradiotherapy; AJCC, American Joint Committee on Cancer; ref, reference group; IMRT, intensity-modulated radiation therapy; 3D-CRT, three-dimensional conformal radiation therapy.

Table S4. Characteristics of patients with TESCC receiving CCRT with IMRT or 3D-CRT and their propensity score–matched cohort.

Variables	All		IMRT		3D-CRT		
	**n = 1076**	**(%)**	n = 538	(%)	n = 538	(%)	*P* value
**Age**							
Mean±SD	58.51	±10.62	58.58	±9.86	58.44	±11.37	0.8289
							1.0000
20-29	4	(0.37)	2	(0.37)	2	(0.37)	
30-39	30	(2.79)	15	(2.79)	15	(2.79)	
40-49	254	(23.61)	127	(23.61)	127	(23.61)	
50-59	338	(31.41)	169	(31.41)	169	(31.41)	
60-69	266	(24.72)	133	(24.72)	133	(24.72)	
70-79	240	(13.38)	72	(13.38)	72	(13.38)	
80-89	144	(3.72)	20	(3.72)	20	(3.72)	
90-100	0	(0.00)	0	(0.00)	0	(0.00)	
**Sex**							1.0000
Male	1026	(95.35)	513	(95.35)	513	(95.35)	
Female	50	(4.65)	25	(4.65)	25	(4.65)	
**Follow-up time (year)**							0.4418
Median, IQR	2.02	1.14	2.04	1.14	2.00	1.14	
(q1, q3)	(0.99,	2.73)	(0.98,	2.77)	(0.99,	2.74)	
(min, max)	(0.09,	6.69)	(0.09,	6.29)	(0.12,	6.96)	
**CCI score**							
mean±SD	1.29	±1.34	1.29	±1.34	1.29	±1.34	1.0000
0	328	(30.48)	164	(30.48)	164	(30.48)	1.0000
1	392	(36.43)	196	(36.43)	196	(36.43)	
2	200	(18.59)	100	(18.59)	100	(18.59)	
>3	156	(14.50)	78	(14.50)	78	(14.50)	
**Radiation dose (Gy)**							1.000
Median, IQR	50.40	±0.21	50.40	±0.21	50.40	±0.22	
**Cumulative cisplatin dose (mg/m^2)**							0.6970
Mean (SD)	253.77	±59.39	254.97	±59.61	253.56	±59.16	
**AJCC clinical stages**							1.0000
Early stage (IA-IIB)	208	(19.33)	104	(19.33)	104	(19.33)	
Advanced stage (IIIA-IIIC)	868	(80.67)	434	(80.67)	434	(80.67)	
**AJCC clinical stages**							1.0000
IA	0	(0.00)	0	(0.00)	0	(0.00)	
IB	14	(1.30)	7	(1.30)	7	(1.30)	
IIA	94	(8.74)	47	(8.74)	47	(8.74)	
IIB	100	(9.29)	50	(9.29)	50	(9.29)	
IIIA	156	(14.50)	78	(14.50)	78	(14.50)	
IIIB	438	(40.71)	219	(40.71)	219	(40.71)	
IIIC	274	(25.46)	137	(25.46)	137	(25.46)	
**Year of diagnosis**							1.0000
2006-2010	696	(64.68)	348	(64.68)	348	(64.68)	
2011-2014	380	(35.32)	190	(35.32)	190	(35.32)	
**Death**							<0.0001
No	214	(19.89)	132	(24.54)	82	(15.24)	
Yes	862	(80.11)	406	(75.46)	456	(84.76)	

CCRT, concurrent chemoradiotherapy; CCI, Charlson comorbidity index; RT, radiotherapy; CCRT, concurrent chemoradiotherapy; AJCC, American Joint Committee on Cancer; ref, reference; IMRT, intensity-modulated radiation therapy; IQR, interquartile range; SD, standard deviation; Gy, Gray; 3D-CRT, three-dimensional conformal radiation therapy.

Table S5. Cox proportional hazard regression model for mortality risk among patients with TESCC receiving CCRT in their propensity score–matched cohort.

Variable	Univariate analysis				Multivariate analysis			
	HR	95% CI		*P*	aHR*	95% CI		*P*
**RT Modalities**								
3D-CRT (ref)	1				1			
IMRT	0.86	(0.78,	0.93)	0.0172	0.85	(0.76,	0.91)	0.00231
**Sex**								
Female (ref)	1				1			
Male	1.03	(0.90,	1.09)	0.9989	1.02	(0.94,	1.08)	0.9990
**Age**								
20-29 (ref)	1				1			
30-39	0.96	(0.61,	10.20)	0.3804	1.02	(0.41,	9.82)	0.5017
40-49	1.05	(0.76,	11.42)	0.3351	1.03	(0.77,	10.50)	0.4846
50-59	1.08	(0.49,	8.15)	0.4599	1.06	(0.51,	8.12)	0.4865
60-69	1.12	(0.67,	7.21)	0.5252	1.11	(0.68,	7.01)	0.6736
70-79	1.13	(0.77,	7.06)	0.5222	1.12	(0.78,	7.04)	0.6362
80-89	1.14	(0.63,	9.92)	0.4955	1.12	(0.67,	9.91)	0.5532
**CCI score**								
0 (ref)	1				1			
1	1.07	(0.84,	1.21)	0.5517	1.04	(0.88,	1.19)	0.6524
2	1.08	(0.85,	1.25)	0.4836	1.14	(0.89,	1.24)	0.6850
>3	1.13	(0.87,	1.42)	0.4289	1.16	(0.91,	1.33)	0.4752
**Year of diagnosis**								
2006~2010 (ref)	1				1			
2011~2014	0.93	(0.87,	1.04)	0.8978	0.91	(0.87,	1.05)	0.8241
**AJCC clinical stages**								
Early stage (ref)	1				1			
Advanced stage	1.28	(0.48,	1.57)	0.5047	1.89	(.51,	1.63)	0.4955

*All variables were used in multivariate analysis. HR, hazard ratio; CI, confidence interval; aHR, adjusted hazard ratio. CCRT, concurrent chemoradiotherapy; CCI, Charlson comorbidity index; RT, radiotherapy; CCRT, concurrent chemoradiotherapy; AJCC, American Joint Committee on Cancer; ref, reference group; IMRT, intensity-modulated radiation therapy; 3D-CRT, three-dimensional conformal radiation therapy.

© 2019 by the authors. Licensee MDPI, Basel, Switzerland. This article is an open access article distributed under the terms and conditions of the Creative Commons Attribution (CC BY) license (http://creativecommons.org/licenses/by/4.0/).
